# Characteristics and Prognostic Implications of High-Risk HPV-Associated Hypopharyngeal Cancers

**DOI:** 10.1371/journal.pone.0078718

**Published:** 2013-11-11

**Authors:** Young-Hoon Joo, Youn-Soo Lee, Kwang-Jae Cho, Jun-Ook Park, In-Chul Nam, Chung-Soo Kim, Sang-Yeon Kim, Min-Sik Kim

**Affiliations:** 1 Department of Otolaryngology-Head and Neck Surgery, College of Medicine, The Catholic University of Korea, Seoul, Korea; 2 Department of Hospital Pathology, College of Medicine, The Catholic University of Korea, Seoul, Korea; Ohio State University Medical Center, United States of America

## Abstract

**Background:**

High-risk human papillomavirus (HPV) is an oncogenic virus that causes oropharyngeal cancers, and it has a favorable outcome after the treatment. Unlike in oropharyngeal cancer, the prevalence and role of high-risk HPV in the etiology of hypopharyngeal squamous cell carcinoma (HPSCC) is uncertain.

**Objective:**

The aim of the present study was to evaluate the effect and prognostic significance of high-risk HPV in patients with HPSCC.

**Methods:**

The study included 64 subjects with HPSCC who underwent radical surgery with or without radiation-based adjuvant therapy. Primary tumor sites were the pyriform sinus in 42 patients, posterior pharyngeal wall in 19 patients, and postcricoid area in 3 patients. High-risk HPV in situ hybridization was performed to detect HPV infection.

**Results:**

The positive rate of high-risk HPV in situ hybridization was 10.9% (7/64). There was a significant difference in the fraction of positive high-risk HPV among pyriform sinus cancer (16.7%), posterior pharyngeal wall cancer (0%), and postcricoid area cancer (0%) (p = 0.042). The laryngoscopic examination revealed a granulomatous and exophytic appearance in 85.7% (6/7) of patients with high-risk HPV-positive pyriform sinus cancer, but in only 31.4% (11/35) of patients with high-risk HPV-negative pyriform sinus cancer (p = 0.012). Significant correlations were found between positive high-risk HPV and younger age (p = 0.050) and non-smoking status (p = 0.017). HPV-positive patients had a significantly better disease-free survival (p = 0.026) and disease-specific survival (p = 0.047) than HPV-negative patients.

**Conclusions:**

High-risk HPV infection is significantly related to pyriform sinus cancer in patients with HPSCC.

## Introduction

Hypopharyngeal squamous cell carcinoma (HPSCC) is the most aggressive malignancy of the head and neck region that occurs in the aerodigestive tract; 70–80% of the cases are found at an advanced stage III and IV because of the aggressive features of the tumor, with a high frequency of nodal metastasis and submucosal extension [1]. Tobacco smoking and excessive alcohol consumption are probably the best known risk factors for HPSCC. However, around 20% of patients with this disease are not exposed to these risk factors, and only a small proportion of individuals who are exposed to tobacco develop squamous cell carcinoma (SCC) of the head and neck [2]. Recently, human papillomavirus (HPV) has been shown to be of prognostic significance, particularly in oropharyngeal SCC. Some studies suggest that HPV, especially types 16 and 18, may be responsible for a small subgroup of oral SCC and up to 50% of oropharyngeal SCC [3], [4]. HPV-positive oropharyngeal SCC seems to be different from HPV-negative oropharyngeal SCC with respect to tumor differentiation, risk factors and the genetic changes that are present, indicating that HPV-positive oropharyngeal SCC represent a separate tumor entity from HPV-negative oropharyngeal SCC, which has specific molecular pathways associated with its tumourigenicity [4], [5]. In contrast to oropharyngeal SCC, little is known about the significance of HPV in other tumor subsites in the head and neck. Specifically, it is unclear whether the correlations found in oropharyngeal SCC can be extrapolated to HPSCC. The aim of the present study was to examine the presence of HPV in the samples of HPSCC. Furthermore, clinicopathological characteristics and disease outcome were correlated with HPV status.

## Materials and Methods

### Ethics statement

The study protocol was approved by the Institutional Review Board of the Catholic Medical Center. Written informed consent was obtained from all patients.

**Table 1:** Demographic profiles and association with incidence of high-risk human papillomavirus.

Parameter	No of patients (%)	No of High risk HPV positive (%)	No of High risk HPV negative (%)	p-value
Age (years)				0.050*
≤60	31 (48.4)	6 (85.7)	25 (43.9)	
>60	33 (51.6)	1 (14.3)	32 (56.1)	
Gender				1.000
Male	63 (98.4)	7 (100.0)	56 (98.2)	
Female	1 (1.6)	0 (0.0)	1 (1.8)	
Smoking status				0.017*
None smoker	18 (28.1)	5 (71.4)	13 (22.8)	
Past smoker	6 (9.4)	1 (14.3)	5 (8.8)	
Current smoker	40 (62.5)	1 (14.3)	39 (68.4)	
Alcohol consumption				0.149
None drinker	28 (43.8)	5 (71.4)	23 (40.4)	
Moderate drinker	10 (15.6)	0 (0.0)	10 (17.5)	
Heavy drinker	26 (40.6)	2 (28.6)	24 (42.1)	
Primary tumor site				0.042*
Pyriform sinus	42 (65.6)	7 (100.0)	35 (61.4)	
Posterior pharyngeal wall	19 (29.7)	0 (0.0)	19 (33.3)	
Postcricoid region	3 (4.7)	0 (0.0)	3 (5.3)	
T classification				0.697
T1/T2	30 (46.9)	4 (57.1)	26 (45.6)	
T3/T4	34 (53.1)	3 (42.9)	31 (54.4)	
Cervical metastasis				1.000
Yes	48 (75.0)	5 (71.4)	43 (75.4)	
No	16 (25.0)	2 (28.6)	14 (24.6)	
Postoperative irradiation				1.000
Yes	44 (68.8)	5 (71.4)	39 (68.4)	
No	20 (31.2)	2 (28.6)	18 (31.6)	
Histologic differentiation				0.796
Well	12 (18.8)	2 (28.6)	10 (17.5)	
Moderately	41 (64.1)	4 (57.1)	37 (64.9)	
Poorly	11 (17.2)	1 (14.3)	10 (17.5)	
Lymphovascular invasion				1.000
Yes	43 (67.2)	5 (71.4)	38 (66.7)	
No	21 (32.8)	2 (28.6)	19 (33.3)	
Extracapsular spread				1.000
Yes	34 (53.1)	4 (57.1)	30 (52.6)	
No	30 (46.9)	3 (42.9)	27 (47.4)	

* p<0.05.

### Patients and tumor samples

The clinical and pathological data of 105 patients who were diagnosed with HPSCC and underwent surgery at the Department of Otolaryngology-HNS, The Catholic University of Korea, Seoul, Korea, from 1996 to 2011, were reviewed retrospectively. The criteria for enrollment were as follows: (1) patients were previously not treated for HPSCC; (2) patients had biopsy-proven SCC; (3) patients received surgery for primary treatment; (4) availability of the original pathological specimens. Patients satisfying at least one of the following criteria were excluded: (1) patients received chemoradiotherapy for primary treatment prior to or as an alternative to surgery; (2) patients being treated for a recurrence of the primary tumor; or (3) patients with distant metastasis at the time of initial presentation. A total of 64 patients met these criteria and were included in the study. The follow-up period ranged from 5 to 126 months, with a mean follow-up period of 32.0 months. At our institution, the initial choice for radical therapy is principally surgery, including partial pharyngectomy, in which neck lymph node metastasis is treated by neck dissection. All patients were treated with primary tumor resection and neck dissection. For tumor excision, 12 (19%) patients underwent partial pharyngectomy with laryngeal preservation, 32 (50%) patients underwent partial laryngectomy with partial pharyngectomy, 14 (22%) patients underwent total laryngectomy with partial pharyngectomy, 5 (8%) patients underwent total laryngopharyngectomy, and 1 (1%) patient underwent total laryngopharyngoesophagectomy. Indications and modalities for adjuvant radio (chemo) therapy varied over time; positive or close margins found on resection, advanced T stage, lymphovascular invasion, perineural invasion, multiple nodal metastasis, or extracapsular spread were the findings that required an additional treatment. Patients underwent laryngoscopic or rigid endoscopic hypopharyngeal examination with photographic documentation before surgery. The patients were staged according to the 2007 edition of the T/N/M classification of the American Joint Committee on Cancer (AJCC).

### In situ hybridization of HPV

Tissue sections (4-μm in thickness) were prepared from formalin-fixed, paraffin-embedded tissues and mounted on 3-aminopropylmethoxysilane-coated slides. In situ hybridization was processed on an automated Benchmark system from Ventana Medical Systems (Tucson, AZ, USA) utilizing INFORM^®^ HPV III Family 16 Probe (cocktail of HPV subtypes 16, 18, 31, 33, 35, 39, 45, 51, 52, 56, and 66, Ventana Medical Systems) as described previously [6], [7]. This system removes the paraffin wax from the tissue, subjects it to protease digestion, and then hybridizes the tissue with a probe. The probe-target complex is detected by the action of alkaline phosphatase on the chromogen nitroblue tetrazolium and bromochloroindolyl phosphate, which yields a dark blue color with a pink counterstain for the HPV-negative cells due to nuclear fast red. Evaluation of the nuclear hybridization signal was performed by a pathologist who had specialized in head and neck pathology (YS Lee).

**Table 2:** Assessment of the clinicopathological factors by the log-rank test.

Parameter	DFS (%)	p-value	DSS (%)	p-value	OS (%)	p-value
Age (years)		0.847		0.764		0.545
≤60	54		62		62	
>60	52		59		52	
Gender		0.677		0.713		0.025*
Male	52		60		57	
Female	100		100		0	
Smoking status		0.384		0.248		0.403
None smoker	71		75		70	
Past smoker	51		54		52	
Current smoker	45		45		45	
Alcohol consumption		0.170		0.151		0.278
None drinker	66		72		66	
Moderate drinker	37		37		37	
Heavy drinker	43		55		52	
Primary tumor site		0.003*		0.049*		0.050*
Pyriform sinus	60		69		67	
Posterior pharyngeal wall	46		43		39	
Postcricoid region	0		0		0	
T classification		0.124		0.335		0.133
T1/T2	65		66		66	
T3/T4	40		52		48	
Cervical metastasis		0.005*		0.024*		0.026
Yes	40		52		48	
No	83		82		82	
Postoperative irradiation		0.082		0.153		0.168
Yes	45		54		52	
No	72		72		66	
Margin involvement		0.201		0.344		0.503
Yes	37		48		46	
No	60		65		60	
Histologic differentiation		0.986		0.633		0.535
Well	57		65		65	
Moderately	45		57		46	
Poorly	39		42		42	
Lymphovascular invasion		0.279		0.470		0.420
Yes	46		54		51	
No	66		68		68	
Extracapsular spread		0.001*		0.003*		0.004*
Yes	29		43		42	
No	76		77		74	
High risk HPV status		0.026*		0.047*		0.324
Positive	100		100		83	
Negative	46		54		52	

DFS, disease-free survival; DSS, disease-specific survival; OS, overall survival.

* p<0.05.

### Statistical analysis

To determine the significance of the association between high-risk HPV and the clinicopathological features, Fisher's exact test, multiple logistic regression analysis, multiple linear regression analysis, and correlation analysis were performed, as appropriate. The survival was determined using the Kaplan-Meier method. The Cox proportional hazards model with likelihood ratio statistics was used to identify variables independently associated with survival. A p<0.05 was considered statistically significant. All calculations were performed using SPSS software ver. 16.0 (SPSS, Chicago, IL, USA).

## Results

### Patient characteristics

The median patient age was 60.6 years (range, 43–80 years); there were 63 males and 1 female. Smoking habit was recorded in all patients: non-smokers (18 patients had never smoked), past smokers (6 patients had stopped smoking at least 1 year before the surgery) or current smokers (40 patients continued to smoke). Alcohol consumption was also recorded: 28 patients were non-drinkers, 10 patients were moderate drinkers (1–3 drinks per week), and 26 patients were heavy drinkers (4–7 drinks per week). The site of the original primary tumor was recorded in all patients and comprised the pyriform sinus in 42 patients, the posterior pharyngeal wall in 19 patients, and the postcricoid area in 3 patients. Regarding the pathological stages, there were 6, 24, 20, and 14 patients with stage T1, T2, T3, and T4 cancers, respectively. Regarding the stage of the cervical lymph nodes, there were 16, 11, 35, and 2 cases in N0, N1, N2, and N3, respectively. Regarding tumor cell differentiation, there was 12, 41, and 11 cases of well- differentiated (G1), moderately differentiated (G2), and poorly differentiated (G3) HPSCC, respectively. Pathologic margin involvement was observed in 15 (23%) of the 64 patients, and 44 patients received postoperative radiotherapy. Lymphovascular invasion was observed in 43 patients, and extracapsular spread of a metastatic neck node was observed in 34 patients.

### Relationship between high-risk HPV positivity and clinicopathological parameters (Table 1)

**Figure 1:**
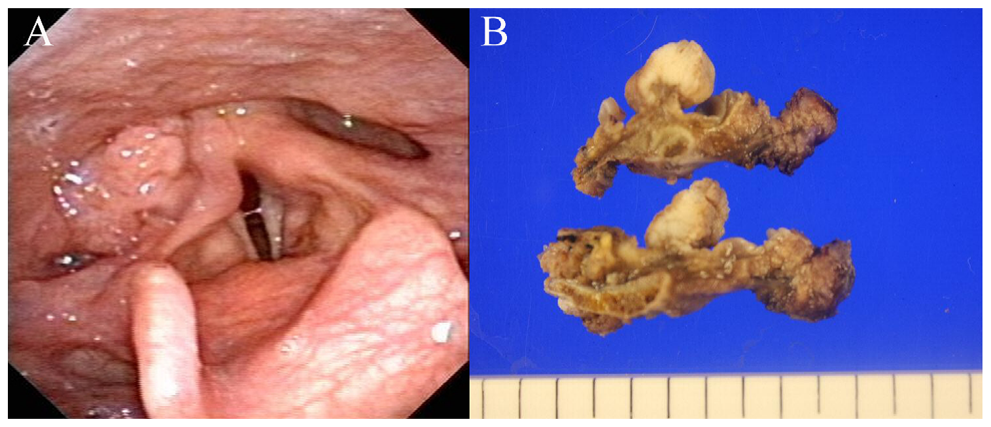
Laryngoscopic view (A) showing left pyriform sinus cancer (arrow) having an exophytic appearance consistent with a high-risk HPV-positive tumor. Vertical sections of the high-risk HPV-positive left pyriform sinus cancer specimens (B).

High-risk HPV (subtypes 16, 18, 31, 33, 35, 39, 45, 51, 52, 56, and 66) was observed in 7 (10.9%) of 64 HPSCC patients. There was a significant correlation between positive high-risk HPV results and primary tumor site (p = 0.042). The rate of positive high-risk HPV test results among the 42 patients with pyriform sinus cancer was 16.7%, whereas that for the 19 patients with posterior pharyngeal wall cancer and 3 patients with postcricoid area cancer was 0%. Laryngoscopic examination of the hypopharyngeal tissue demonstrated that 6 of the 7 patients (85.7%) with high-risk HPV-positive pyriform sinus cancer showed a granulomatous and exophytic appearance, but only 11 of the 35 patients (31.4%) with high-risk HPV-negative pyriform sinus cancer showed this appearance (p = 0.012) (Fig. 1). In addition, younger age and non-smoking status were associated with positive high-risk HPV findings in HPSCC (p = 0.050 and p = 0.017, respectively). However, there was no significant relationship between gender, alcohol consumption, T stage, cervical lymph node metastasis, tumor cell differentiation, lymphovascular invasion, extracapsular spread and high-risk HPV. The multivariate analysis showed that only the non-smoking status was significantly correlated with high-risk HPV positivity (p = 0.015). The odds ratio was 18.02 (95% confidence interval [CI], 1.76–184.11) for non-smokers compared with current smokers.

### Relationship between high-risk HPV positivity and survival rate

**Figure 2:**
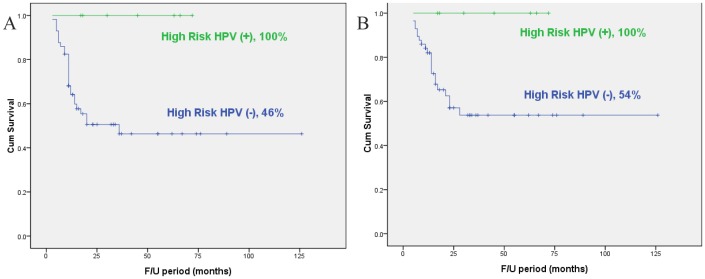
Kaplan–Meier disease-free survival curve (A) and disease-specific survival curve (B) according to the high-risk HPV status.

The 5-year disease-free survival (DFS), disease-specific survival (DSS), and overall survival (OS) rate in our cohort were 52%, 60%, and 57%, respectively. The patients with high-risk HPV negativity had a significantly worse 5-year DFS (p = 0.026) and DSS (p = 0.047) as compared with patients with high-risk HPV positivity according to the Kaplan-Meier survival curves (Table. 2) (Fig. 2). On the other hand, primary tumor site, cervical lymph node metastasis and extracapsular spread were found to be associated with 5-year DFS, DSS, and OS by univariate analysis. Multivariate Cox regression analysis confirmed the significant association between extracapsular spread and 5-year DFS (hazard ratio [HR], 4.70; 95% CI, 1.88–11.75; p = 0.001) and DSS (HR, 4.50; 95% CI, 1.63–12.43; p = 0.004). Details of the multivariate analysis are provided in Table 3.

**Table 3:** Cox proportional hazards analysis for survival at 5 years.

Parameter	Disease Free Survival		Disease Specific Survival		Overall Survival	
	Hazard Ratio	p-value	Hazard Ratio	p-value	Hazard Ratio	p-value
Male	0.32	0.571	0.25	0.616	2.64	0.104
Primary tumor site	3.40	0.183	0.99	0.609	2.72	0.257
Cervical metastasis	1.46	0.227	0.57	0.451	0.73	0.394
Extracapsular spread	4.70	0.001*	4.50	0.004*	3.56	0.008*
High risk HPV negativity	5.82	0.970	4.82	0.973	1.59	0.208

* p<0.05.

## Discussion

The hypopharynx extends from the superior border of the epiglottis and the pharyngoepiglottic folds from the hyoid bone to the lower border of the cricoid cartilage. It is divided into three primary anatomic subsites: the pyriform sinuses, the postcricoid area, and the posterior pharyngeal wall [8]. HPSCC is a highly aggressive cancer that is generally diagnosed at an advanced stage, and consequently it has a poor prognosis and a low survival rate. Multidisciplinary treatment including surgery, radiotherapy, and chemotherapy has been applied to treat this tumor, and it has improved the prognosis of the patients [9]–[11]. In this study, we investigated the correlation between the high-risk HPV and its prognostic significance in patients with HPSCC who were treated with radical surgery with or without adjuvant therapy. Although a slightly higher proportion of patients with high-risk HPV-positive tumors received adjuvant radiation (71%, 5 out of 7 patients) compared to those with high-risk HPV-negative tumors (68%, 39 out of 57 patients), the 5-year DSS rate increased dramatically with high-risk HPV positivity (100% vs 54%). High-risk HPV-negative tumors appear to be more aggressive with or without adjuvant radiotherapy. The patients with high-risk HPV- positive HPSCC had a 100% DFS and DSS. However, only small patients with high-risk HPV- positive HPSCC treated with primary surgery were included in this study. This result needs to be tested in a larger numbers of HPSCC patient treated with surgery or chemoradiation.

In an international collaborative study in eight geographic areas, the seroprevalence against HPV virus-like particles and HPV DNA for high-risk HPV16 and HPV18 was relatively low in Korea [12]. Although the prevalence of HPV infection varies according to the age groups, geographic areas, and detection methods; the reported overall prevalence of HPV infection is 10–15% [13]. We previously reported that the prevalence of HPV infection in oral and oropharyngeal cancers was 6.7% and 27.3%, respectively [6]. In this study, the positive rate of high-risk HPV in situ hybridization was 10.9%. Shin et al reported that HPV-related oropharyngeal cancer had increased significantly over the period 1999–2009, particularly in young men, whereas HPV-unrelated sites such as larynx and hypopharynx, decreased markedly in both sexes [14]. Over the past several decades, Korea has experienced rapid socioeconomic growth; it has one of the highest growth rates in the world. There are rapid changes in the lifestyle, including sexual behavior in Korea, from a conservative Asian lifestyle to a more open Western lifestyle. Thus, a better understanding of the impact of HPV infection and associated cancers of the head and neck will be needed in order to manage this growing population effectively.

In the present study, we demonstrated that HPSCC in the pyriform sinus were more likely to be HPV-related than in other hypopharyngeal subsites. The pyriform sinus cancer arises from the mucosa of the anatomic subsite of the hypopharynx represented as analogous to an inverted pyramid situated lateral to the larynx, with the base located superiorly and the anterior, lateral, and medial walls narrowing inferiorly to form the apex with its tip extending slightly below the cricoid cartilage [8]. The exact mechanism of HPV infection in the hypopharyngeal region remains uncertain, but the easy access to the pyriform sinus and favorable microenvironmental factors may be the causes of the high frequency of HPV identified in the pyriform sinus. We also observed that HPV infection status was related to the characteristic gross features of the tumors. Similar to HPV-related oropharyngeal cancers, HPV-positive pyriform sinus cancer showed a tendency for granulomatous and exophytic growth, whereas HPV-negative pyriform sinus cancer tended to demonstrate ulcerative or flat growth.

There were a few limitations to this study. The main limitation was that a retrospective analysis was employed. Second concern was selection bias in that only patients treated with primary surgery were included. Third, patients with HPSCC at different subsites and T stages were not equally distributed. Fourth, we were unable to perform testing of the HPSCC tissue for HPV using PCR-based assays. Lastly, the median follow-up period of this study was relatively short. It was thought that there were limitations of the DFS and DSS data. Future prospective, randomized studies assessing oncologic results following different treatment modalities are required to address these issues.

In conclusion, the results presented in this study suggest that high-risk HPV infection is more likely to be associated with pyriform sinus cancer than cancer at other subsites in patients with HPSCC. On the basis of our results, we believe that high-risk HPV infection is a significant predictor of reduced recurrence and prolonged survival. An adequate assessment of high-risk HPV status can provide valuable prognostic information in patients with HPSCC.
